# Idiopathic pulmonary fibrosis: the need for early diagnosis

**DOI:** 10.1186/2049-6958-8-53

**Published:** 2013-08-09

**Authors:** Gaetano Cicchitto, Claudio M Sanguinetti

**Affiliations:** 1 RespiratoryPhysiopathology Unit, ASL SA, Cava de’ Tirreni, SA, Italy; 2 Senior Consultant in Respiratory Medicine, Editor and Managing Director of Multidisciplinary Respiratory Medicine, Rome, Italy

**Keywords:** Biomarkers, Early diagnosis, Idiopathic pulmonary fibrosis, Omics

## Abstract

Idiopathic pulmonary fibrosis (IPF), a chronic fibrosing lung disease of a progressive nature and unknown etiology, has the largest epidemiological impact and the worst prognosis among the idiopathic interstitial pneumonias (IIP). Despite the progress in pathogenetic knowledge, many aspects are still dubious, in particular the biomolecular mechanisms activated in the early stages of the disease. Early diagnosis is desirable not only to better define aspects of the natural history of the disease, but also to customize treatment protocols. An early diagnosis of IPF should necessarily be based on the ability to highlight a number of features drawn not only from a careful composition of specific anamnestic data with clinical, functional and radiological parameters, but also from biological markers that, in a proper context, can provide guidance and confirm a clinical-anamnestic suspicion. The identification of specific biomarkers for IPF is a modern and attractive look for the potential clinical implications in terms of diagnosis, prediction of disease progression and prognosis. Biomolecular investigations on IPF were performed selectively on tissue samples, bronchoalveolar lavage (BAL), or blood: nowadays the “multi-omic” approach may allow studying individual constitutional profiles resorting to a series of biomolecular disciplines, the so-called “omics”, which focuses on responses of the entire genomic complex, in line with the current trend to quantitatively analyze the interactions of all components of a biological system. Such refined investigations are an essential base for research now, but they might become a routine in the near future, allowing a more precise classification of patients suffering from a disease of unclear taxonomy.

## Review

Idiopathic pulmonary fibrosis (IPF) is a chronic fibrosing lung disease, of a progressive nature, unknown etiology, limited to the lung, which, in the context of the idiopathic interstitial pneumonias (IIP), has the largest epidemiological impact and the worst prognosis [1]. Epidemiological data still qualify IPF as a “rare disease”, even if international studies seem to report an increasing incidence [2,3]. It is uncertain, however, whether this depends on the aging of the population, changes in smoking habits, environmental exposure to pneumotoxic substances, greater awareness and possibilities to make a diagnosis of the disease, or a combination of these factors [4].

Unlike other pulmonary diseases (Chronic Obstructive Lung Disease (COPD), neoplasms), neither risk factors nor natural history are clear for IPF. In addition, despite the progress in pathogenetic knowledge, many aspects are still dubious; among these, in particular the biomolecular mechanisms activated in the early stages of the disease. The most significant consequence of such uncertainty is a delay in clinical suspicion, so that diagnosis is usually made when clinical objectivity and anathomo-radiological alterations have already achieved an irreversible expression.

The growing incidence and an inexorable progression of IPF to functional impairment have not only directed research towards the understanding of the pathogenesis and molecular bases of the disease [5,6], but also encouraged the academic community to propose a number of therapeutic trials [7], particularly in the last years. These trials, however, have not provided solid and unequivocal efficacy [8], with one possible exception for pirfenidone [9], on the real usefulness of which, however, a general agreement has not been reached yet [10]. The variability of the clinical course in each patient and the difficulty to set up and properly assess clinical trials [11] explain, at least partially, the lack of consistency of the results of such trials and the non-uniformity of interpretation of the data one can find in literature. In this context, it is notable the unavailability of trials involving the initial stages of the disease, *i.e*. when the variable pathobiological processes in progress could be, at least in theory, reversible.

Early diagnosis is, therefore, desirable not only to better define some aspects of the natural history of the disease, but also to customize treatment protocols that at this time are inevitably limited to the above mentioned clinical trials. This in order to optimize the clinical management of patients, who are still faced with a particularly unfavorable prognosis in the short and medium term, as demonstrated by several survival studies [12].

However, the early diagnosis of IPF is not easy for a number of reasons. A first difficulty is the non-specificity of clinical symptoms at onset (non-productive cough, exertional dyspnea). It was observed that 1 to 3 years may elapse between the onset of symptoms and the specialist’s assessment, while the delay in diagnosis can even increase up to 5 years [13]. Functional evaluations do not add to specificity of clinical suspicion: a restrictive ventilatory defect, and a reduction in lung volumes in particular, as well as an alteration of the diffusing capacity of the lung for carbon monoxide (DL_CO_), are a common feature of various interstitial lung diseases (ILD). In addition, lung volume can remain unaltered if there is a concomitant emphysema [14], an association that can be justified by smoking exposure, which represents a common risk factor for both disorders.

Regarding imaging aspects, high-resolution chest tomography (HRCT) is the method of choice to highlight a pattern of usual interstitial pneumonia (UIP), the pathological-radiological substrate of IPF, the detection of which may be based on the distinguishing feature of honeycombing [1]. Unfortunately, this aspect is difficult to grasp until some more overt alterations appear, such as the reticular pattern at the bases and in the periphery of the lung, the presence of micro-cysts, the scarcity of ground glass*,* aspects, etc. [15].

In the light of the foregoing issues, an early diagnosis of IPF should necessarily be based on the ability to highlight, in the very early phases of the disease, a number of features drawn not only from a careful composition of specific anamnestic data with clinical, functional and radiological parameters, but also from biological markers *(*biomarkers*)* that, in a proper context, can provide guidance and confirm a clinical-anamnestic suspicion.

In general, a biomarker indicates a measurable biological characteristic (*e.g.* the concentration of a protein in a biological fluid, a specific functional parameter, a particular morphological expression in HRCT, and so on) at a certain time in a given disease, correlated with the presence, the progression and/or therapeutic responsiveness of the disease [16].

The identification of specific biomarkers for IPF is a modern look and attractive for the potential clinical implications in terms of diagnosis, prediction of disease progression and prognosis. An ideal biomarker should be easily accessible, measurable and suitable to be used for longitudinal assessment [17]. The need of having such indicators available is related to several purposes. First, to overcome limitations arising from the current diagnostic criteria: radiological aspects are not only late, but also sometimes not univocal in interpretation. On the other hand, surgical biopsy is very often not feasible for both the poor “performance status” of patients and their refusal to undergo a surgical procedure. In any case, a special expertise is requested for radiological and surgical procedure and interpretation, only possible in reference centers. Secondly, the availability of biomarkers repeatable over time can improve the clinical management of the patients, making it possible, for example, to provide prognostic information and optimize the inclusion in the lists of lung transplantation.

The identification and mapping of the human genome have led to the introduction of increasingly sophisticated analytical methods and the emergence of new molecular disciplines (genomics, transcriptomics, proteomics, metabolomics: omics). Accordingly, a re-profiling of various respiratory diseases, including IPF, took place within a molecular approach. In this regard a biological marker can be defined as any expression of processes involving cells (proteins, metabolites, etc..) or of a gene that can transmit information on the state of health or disease of an individual and that, depending on the type of new information provided, can be used in a specific clinical setting (diagnosis, susceptibility, prognosis, etc.) [18].

Acquisitions of genomics may be of heterogeneous origin. The substrate most used, at least originally, was of course the lung tissue of IPF patients, obtained from lung biopsy, autopsy or explant [19].

In one study Selman et al. [20], employing microarrays techniques, showed a different gene expression profile in IPF compared to other chronic fibrosing lung diseases, in particular the hypersensitivity pneumonitis (HP). In a subsequent work [21] they reported a distinct biological and transcriptional structure in patients with “slow” and “progressive” forms of IPF.

Boon et al. [22], using a serial analysis of gene expression (SAGE), have confirmed a different gene expression in patients with IPF compared to healthy subjects or to patients with other respiratory conditions, and between “stable” and “accelerated” phenotypes in the same IPF. In addition, these AA [22] observed that some gene products related to progression of the disease were also found in biological fluids (blood and/or BAL).

Konishi et al. [23], still using microarray analysis, have emphasized a “molecular signature” distinguishable in acute exacerbations of IPF (AEIPF), and have observed that, in cases of AEIPF, the concomitant finding of an increased level of α-defensins in the blood may envisage a clinical role for these peptides as biomarkers to be used for patients monitoring.

The regulation of gene expression is a particularly complex aspect, being able to modulation through not only a transcriptional and post-transcriptional control, but also an epigenetic one. Epigenetic is defined as the study of changes in transcriptional profile that do not involve changes in the DNA sequence, often in response to environmental stimuli, and heritable through cell generations [24]. The potential reversibility [25] of these events explains the growing interest in the possible therapeutic implications. Epigenomic analysis basically is based on evaluation of DNA methylation, histone modifications and the expression of microRNAs (miRNAs) [26]. Extensive evidence supports the importance of epigenetic mechanisms in the pathogenesis of IPF.

Sanders et al. [27] found no significant differences in global DNA methylation between normal and IPF lung, but for IPF they reported an altered activity of some enzymes methylating in specific anatomical regions (in particular, fibroblastic foci), which corresponded to an altered pattern of messenger RNA (mRNA), demonstrating the involvement of a number of disrupted genes.

Pandit et al. [28] have summarized the available data for miRNAs, short non-coding RNA molecules involved in post-transcriptional gene regulation and often associated with tissue dysfunction. In IPF a fundamental pathogenetic moment of the fibrosing process seems to be the epithelial-mesenchymal transition (EMT), a biological phenomenon that different miRNAs can promote (*e.g.* miR-2 [29], miR-155 [30]) or inhibit (*e.g.* Let-7d, miR-29) [31].

The importance of these observations is not limited to the understanding of the pathogenetic aspects only, since they may make possible, at least theoretically, to envisage a therapeutic strategy polarizing towards miRNAs with an antifibrosing action [32].

Epigenetic regulation, however, may act through multiple mechanisms and interferences. An *in vitro* study described the inhibition of histone deacetylation as a possible factor for a decrease in survival of lung fibroblasts from patients with IPF, resistant to apoptosis, through modifications of DNA methylation [25]. Dakhlallah et al. [33] identified in IPF a complex interactive circuit between an aberrant DNA methylation and the regulation of expression of the cluster MiR-17 ~ 92. In addition, a pharmacological modulation causing a re-expression of this gene cluster seemed to reduce the genes’ fibrosing potential.

Feasibility of such sophisticated strategy is, of course, conditional upon the availability of lung tissue obtained by way of surgical biopsy, with the consequent limitations inherent to such an approach, in particular sampling variability (sampling bias) [34] and risk of AEIPF [35]. Other aspects should not be overlooked, such as the presence of comorbidities, in a disease that typically affects the elderly, and the mutability of interpretation of morphological patterns. This generates the need to make use of so-called “surrogate tissue”, i.e. biological material obtained through partially invasive methods (blood, BAL) [19].

The search for markers in biological fluids substantially relies on serological analyses which led to the identification of various molecules. Following the current guidelines relating to the main pathogenetic biological elements involved, such molecules may be divided, according to their origin, into two categories: the compounds derived from type II pneumocytes and those derived from macrophages [36]. More recently, substances from the extracellular matrix have also been identified as potential biomarkers [37].

Surfactant proteins A and D (SP-A, SP-D) are lipoprotein complexes, synthesized by type II pneumocytes (AEC II) and by the Clara cells, mainly active in the reduction of the surface tension, but also functioning as a defense in the context of natural immunity. An increase in SP-A and SP-D [38] in serum from IPF patients, was linked to a presumed increase in the synthesis by hyperplastic AEC IIs or to transudation resulting from decomposition of the epithelium and basement membrane, and this increase was correlated with mortality. These studies seem therefore to suggest that SP-A and SP-D may be potential diagnostic and prognostic biomarkers [39].

Another molecule derived from respiratory epithelium (AEC II and bronchiolar cells) is the Krebs von den Lungen 6 Antigen (KL6) glycoprotein, a factor of fibroblast proliferation and survival [40,41], whose levels resulted high in both serum and BAL fluid [42] from patients with various interstitial lung diseases (ILD) of fibrosing character, both idiopathic (such as IPF and NSIP [43]) and associated with collagen-vascular disease [44]. Since serum levels > 1000 U/ml seem to be correlated with survival, measuring KL6 may have a prognostic significance in both the IIP and lung fibroses associated with collagen-vascular diseases [45].

The chemokine CCL18, synthesized by alveolar macrophages polarized towards the M2 phenotype [46], exerts a chemotactic action on fibroblasts, stimulating their collagen production [47]: CCL18 values > 150 ng/ml resulted predictive of mortality [48], which gives a useful prognostic value to this marker.

The chemokine CCL2, produced by various cells including macrophages, while being correlated with the presence of pulmonary fibrosis in general and with the clinical course in IPF [43,49], was increased also in other diseases so limiting its role as a biomarker.

The glycoprotein YKL-40, present in macrophages and epithelial cells, belongs to the family of chitinases and it regulates the growth and survival of mesenchymal cells [50], including fibroblasts. Elevated levels of YKL-40 were detected in both serum and BAL fluid of IPF patients [50,51] and resulted correlated with survival, so that it might be a useful prognostic marker.

Calgranuline B (S100A9), expressed in macrophages and neutrophils, was detected at an abnormally high level in IPF compared to controls and other ILDs; it has therefore been proposed as a diagnostic biomarker [52].

The matrix metalloproteinases (MMPs) constitute a family of zinc-dependent endoproteases, involved in the degradation of the extracellular matrix, but also in the processing of many bioactive molecules. Rosas et al. [37] showed that a combined increase in serum MMP1 and MMP7 was discriminating between IPF and other ILDs or COPD, which suggested for these molecules a diagnostic role and, for MMP7 only, also a prognostic one, so that Richards et al. [53] have developed a mortality-predictive multidimensional index involving integration of plasmatic MMP7, FVC and DL_CO_.

In addition to molecular compounds, cellular elements have been proposed as biomarkers, in particular circulating fibrocytes, i.e. cells derived from bone marrow able to develop a mesenchymal differentiation (fibroblasts and myofibroblasts), and Moeller et al. [54] underlined that an increase in fibrocytes> 5% testifies a poor prognosis. A role as biomarkers has been suggested also for additional proteins (osteopontin, periostin) involved in the pathogenesis of IPF [17].

From the above findings in the literature one may easily gather that, regardless of etiology, various biological factors are involved and interact in the process of chronic remodeling and fibrosis of the lung: these factors can therefore potentially be used as biomarkers in both ILD [55] in general and particularly in IPF [17]. Concerning IPF, numerous studies have been conducted using biomolecular analyses to clarify various aspects of clinical significance. The objectives of such works were, however, directed to the study of pathogenetic mechanisms [56], in order to identify new therapeutic “targets”, to diagnostic characterization of the specific phenotypes [22] (e.g. slow vs. fast progressors) of IPF, to the identification of the presence, extension [57] or prognosis [53] of the disease, or, finally, to describe a “pattern” of gene expression [20,23,58]. Biomolecular investigations on IPF were performed selectively on tissue samples, BAL, or blood, in a diagnostic context which had already been defined, and not with the aim of reaching an early diagnosis.

## Conclusions

In conclusion, IPF is a progressive and fatal fibrosing lung disease, caused by a complex and only partially known interaction between exposure to pneumotoxic agents and predisposing conditions. The “multi-omic” approach may allow studying individual constitutional profiles resorting to a series of biomolecular disciplines, the so-called “omics”, which provide various methods that are, at least in principle, complementary. Such an approach implies a shift from a “gene-centric” vision of IPF, in which a given phenotype is believed to derive from the response of a single gene to injury, to a global genome (“genome-wide”) vision, which focuses on responses of the entire genomic complex [19], in line with the current trend to quantitatively analyze the interactions of all components of a biological system [59] in order to define the phenotype of various lung conditions (“systems biology”).

In an age of “integrated” approach to respiratory diseases [60], made possible by implementation of biomolecular analytical technologies, and of “holistic” management of IPF [61], the use of biological markers may be extended from the search for molecules in blood to the identification of gene alterations [62] (single nuclear polymorphisms: SNP, gene mutations), as well as epigenetic ones [26] and changes in particular substances [63]. IPF, therefore, does not appear as a disease from an alteration of a single gene, although single mutations were found, such as those concerning the coding of the telomerase enzyme complex [64] or SNP, in particular in the gene encoding mucin 5B (MUC5B) [65], measurable on blood and BAL as well. These changes, however, may be regarded not as an expression of disease, but rather as an increased predisposing condition, while more significant clinical information could result from methods allowing accurate analyses of genetic and epigenetic expression [34,35] (e.g., miRNA, methylation). On the other hand proteomics research, being able to recognize specific protein clusters (MMP1 and 7, SPP1, YKL-40, etc.) [17], could complement genetic investigation and contribute to the building of a biological “profile” of the pathology: many of the current obstacles may be overcome through increased access to data from the Lung Tissue Research Consortium [66], founded by the National Health Institute with the aim of collecting biological and clinical material concerning respiratory diseases, particularly COPD and ILD.

Such refined investigations, which seem rather futuristic, are an essential base for research now, but they might become a routine in the near future, allowing a more precise classification of patients suffering from a disease that is at present so disappointing as to therapeutic response and prognostic expectation.

## Competing interests

The authors declare that they have no competing interests.
